# 2400. Compared Performance Indices of the 2023 Duke-ISCVID, the 2000 Modified Duke, and the 2015 ESC Criteria for the Diagnosis of Infective Endocarditis

**DOI:** 10.1093/ofid/ofad500.2020

**Published:** 2023-11-27

**Authors:** François Goehringer, Benoît Lalloué, Christine Selton-Suty, François Alla, Guillaume Baronnet, Elisabeth Botelho-Nevers, Catherine Chirouze, Elodie Curlier, Safwane El Hatimi, Marie-Line Erpelding, Lelia Escaut, Amandine Gagneux-Brunon, Mesut GUN, Benjamin Lefèvre, Vincent Le Moing, Lionel Piroth, Aleyya Radjabaly Mandjee, Thibault Sixt, Christophe Strady, Noémie Tissot, Christophe Tribouilloy, Jean-Marc Virion, Nelly Agrinier, Xavier Duval, B R U N O HOEN

**Affiliations:** Nancy University Hospital, Nancy, France, vandoeuvre Lès Nancy, Lorraine, France; CHRU-Nancy, INSERM, Université de Lorraine, CIC, Epidémiologie Clinique, Vandœuvre-lès-Nancy, Lorraine, France; University Hospital of Nancy, Nancy, Lorraine, France; Chu de Bordeaux, Bordeaix, Aquitaine, France; Nancy University Hospital, Vandoeuvre les Nancy, Lorraine, France; Service d’infectiologie, CIC1408, Inserm, CHU de Saint-Etienne, 42055 Saint-Etienne, France; F-CRIN, I REIVAC/COVIREIVAC, France, Saint Etienne, Auvergne, France; Chrono-environnement UMR6249, CNRS, Université Bourgogne Franche-Comté, F-25000, Besançon, France, Besançon, Franche-Comte, France; University Hospital of Besançon, Besançon, Franche-Comte, France; Cardiology Bicetre hospital, AEPEI, Le Kremlin-Bicêtre, Ile-de-France, France; CHRU-Nancy, INSERM, Université de Lorraine, CIC, Epidémiologie Clinique, 54000, Nancy, France, Vandoeuvre-Les-Nancy, Lorraine, France; Infectious diseases Bicêtre hospital, Le Kremlin Bicêtre, Ile-de-France, France; University Hospital of Saint-Etienne, Saint-Etienne, Rhone-Alpes, France; CHU AMIENS, Amiens, Picardie, France; Université de Lorraine, APEMAC && CHRU-Nancy, Nancy, France, Vandoeuvre-Lès-Nancy, Lorraine, France; CHU de Montpellier, Montpellier, Languedoc-Roussillon, France; CHU Dijon, dijon, Bourgogne, France; Universite de Lorraine, CHRU-Nancy, Infectious and Tropical Diseases, Nancy, France, Nancy, Lorraine, France; Dijon University Hospital, Dijon, Bourgogne, France; Groupe Courlancy-Reims, REIMS, Champagne-Ardenne, France; Besançon hospital center, BESANCON, Franche-Comte, France; Université Picardie Jules vernes france, Amiens, Picardie, France; CIC 1433 Epidémiologie Clinique, Vandœuvre-lès-Nancy, Lorraine, France; Université de Lorraine, Inserm, CHRU Nancy, CIC- Epidémiologie clinique, Nancy, Lorraine, France; AP-HP, Bichat Hospital; University of Lorraine at Nancy, France, Vandoeuvre Les Nancy, Lorraine, France

## Abstract

**Background:**

The 2023 Duke-ISCVID criteria for infective endocarditis (IE) were recently proposed to update the diagnostic classification of IE. Using an open prospective multicenter cohort of patients treated for IE (ObservatoireEI, NCT03272724), we evaluated the performance indices of these new criteria, compared with those of the 2000 Modified Duke and the 2015 ESC criteria.

**Methods:**

Data of patients who developed IE between January 2017 and October 2022, were extracted from the cohort database. Each case was individually adjudicated by 3 IE expert clinicians who met to identify cases they deemed ‘certain IE', which formed the “gold standard” IE group. A case was adjudicated as a ‘certain IE’ only when all 3 experts agreed on certainty of IE. Within each classification, each case was summarized by its criteria and assigned one of the definite, possible, or rejected categories. For each diagnostic classification, sensitivity, specificity, and accuracy and their 95% confidence intervals were computed in the whole case sample and in selected sub-groups (Figure).

Case distribution of the 1194 cases by the three sets of criteria

**Figure:**
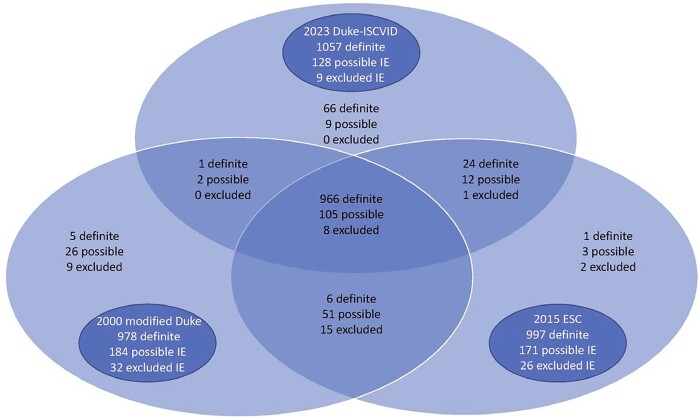

**Results:**

Cases included 1194 patients (mean age 66.1 years, 71.2% men), of whom 680 (57%) had a previously known pre-existing heart disease (414 prosthetic valves), 284 (23.8%) had a CIED, and 94 (7.9%) had a prior history of IE (see additional characteristics in Table 1).

Of the 1194 patients, 946 (79.2%) were adjudicated as certain IE; 978 (81.9%), 997 (83.5%), and 1057 (88.5%) were classified as definite IE in the 2000 modified Duke, the 2015 ESC, and the 2023 Duke-ISCVID criteria, respectively (Figure). In the whole case sample, the sensitivity of each classification was 93.2% [91.6%; 94.8%], 95.0% [93.7%; 96.4%], and 97.6% [ 96.6%; 98.6%], respectively (p< .001 for all 2-by-2 comparisons). Corresponding specificity rates were 61.3% [55.2%; 67.4%], 60.5% [54.4%; 66.6%], and 46.0% [39.8%; 52.2%], respectively. Corresponding accuracy rates were 86.6% [84.7%; 88.5%], 87.9% [86.0%; 89.7%]), and 86.9% [84.9%; 88.8%], respectively. Performance indices on selected subgroups of patients are shown in Table 2.

**Figure:**
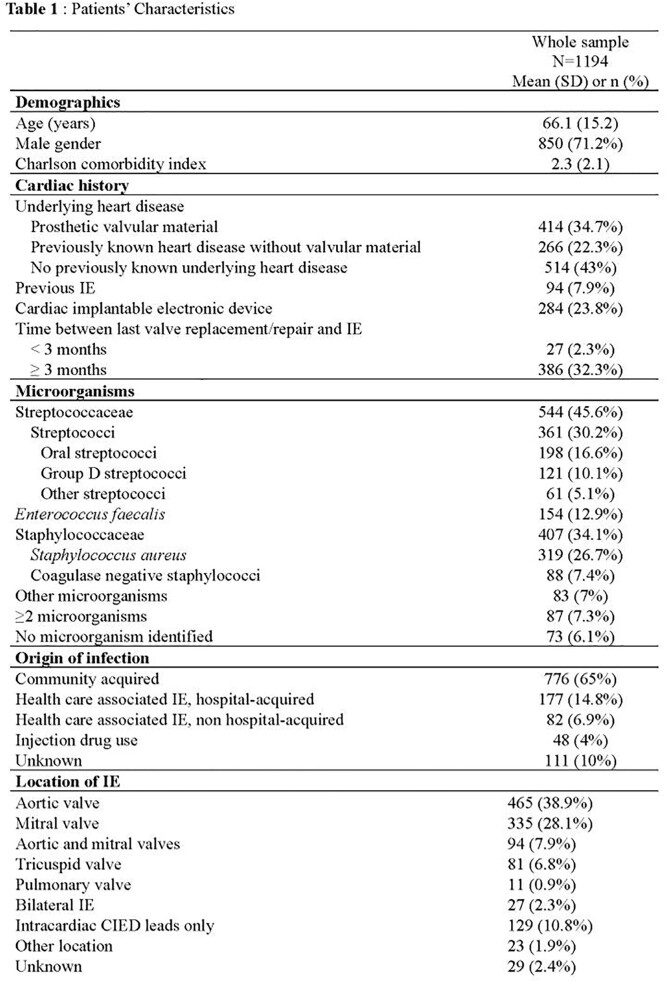

Patients’ Characteristics (Cont'd)

**Figure:**
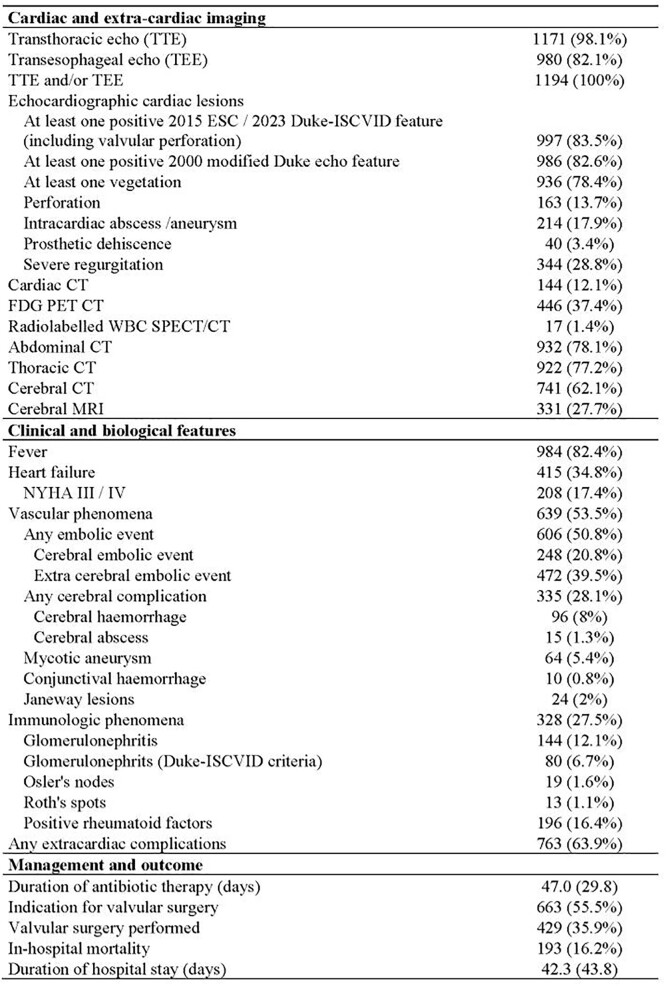

Performance indices (% and [95% CI]) of the 2000 modified Duke, 2015 ESC, and 2023 Duke-ISCVID criteria for the diagnosis of IE in the whole study sample and selected subgroups

**Figure:**
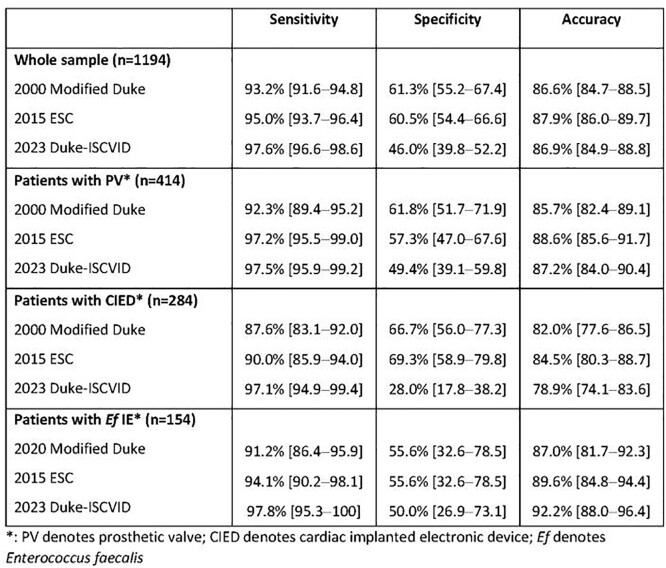

**Conclusion:**

Compared to the 2000 Modified Duke and the 2015 ESC criteria, the 2023 Duke-ISCVID criteria had a significantly higher sensitivity and a significantly lower specificity, while accuracy was not significantly different.

**Disclosures:**

**françois Goehringer, n/a**, Gilead Sciences: Expert Testimony|Gilead Sciences: Honoraria|GSK: Expert Testimony **Christophe Strady, n/a**, shionogi: Honoraria

